# Potential Diagnostic and Prognostic Biomarkers for Adenovirus Respiratory Infection in Children and Young Adults

**DOI:** 10.3390/v13091885

**Published:** 2021-09-21

**Authors:** Giovanni Battista Biserni, Sara Scarpini, Arianna Dondi, Carlotta Biagi, Luca Pierantoni, Riccardo Masetti, Sugitha Sureshkumar, Alessandro Rocca, Marcello Lanari

**Affiliations:** 1 Specialty School of Pediatrics, Alma Mater Studiorum, University of Bologna, 40126 Bologna, Italy; giovanni.b.biserni@gmail.com (G.B.B.); sscarpini@gmail.com (S.S.); 2 Pediatric Emergency Unit, Scientific Institute for Research and Healthcare (IRCCS), Azienda Ospedaliero—Universitaria di Bologna, 40138 Bologna, Italy; carlotta.biagi@aosp.bo.it (C.B.); luca.pierantoni@aosp.bo.it (L.P.); alessandro.rocca4@unibo.it (A.R.); marcello.lanari@unibo.it (M.L.); 3 Pediatric Unit, Scientific Institute for Research and Healthcare (IRCCS), Sant Orsola Hospital, 40138 Bologna, Italy; riccardo.masetti5@unibo.it; 4 Institute of Global Health, University of Geneva, 1205 Geneva, Switzerland; Sugitha.Sureshkumar@etu.unige.ch

**Keywords:** adenovirus, acute upper respiratory tract infections, pneumonia, inflammatory markers, children

## Abstract

Human Adenoviruses (HAdV) are known to be potentially associated with strong inflammatory responses and morbidity in pediatric patients. Although most of the primary infections are self-limiting, the severity of clinical presentation, the elevation of the white blood cell count and inflammatory markers often mimic a bacterial infection and lead to an inappropriate use of antibiotics. In infections caused by HAdV, rapid antigen detection kits are advisable but not employed routinely; costs and feasibility of rapid syndromic molecular diagnosis may limit its use in the in-hospital setting; lymphocyte cultures and two-sampled serology are time consuming and impractical when considering the use of antibiotics. In this review, we aim to describe the principal diagnostic tools and the immune response in HAdV infections and evaluate whether markers based on the response of the host may help early recognition of HAdV and avoid inappropriate antimicrobial prescriptions in acute airway infections.

## 1. Introduction

Human Adenoviruses (HAdVs) are DNA viruses of the Adenoviridae family, which can produce many different clinical pictures during childhood.

Infections are almost always self-limiting in immunocompetent patients, but the severe systemic presentation combined with laboratory alterations such as elevation of white blood cell (WBC) count with neutrophilic prevalence and of C-reactive protein (CRP) may suggest a bacterial infection. This may result in hospital admission and inappropriate use of antibiotics, with an increased risk of potential side effects, antimicrobial resistance, and ensuing human and economic burden [1,2,3].

The aim of the present paper is to provide an overview of immunological responses of adenoviral infections that may add useful information for diagnosis.

### 1.1. Viral Structure and Classification

HAdVs are double-stranded DNA viruses that belong to the Adenoviridae family. They are non-enveloped viruses with a diameter of up to 90 nm, structured with an external icosahedral capsid. The capsid facet is made of hexons with pentons at the vertices, each of them with a fiber for receptor binding. The capsid contains the core, which holds the viral DNA genome with histone-like proteins [4].

Currently, more than 100 different types of HAdVs have been identified, classified into seven species (A–G), and marked with consecutive numbers. Their number is increasing thanks to homologous recombination (mainly for species D, the largest one, including more than 70 different types) and mutation events (especially for species B) [5,6]. Until the discovery of the 51st type, HAdVs were characterized by serum neutralization and hemagglutination inhibition assays, but recently genomic and bioinformatic analyses have replaced serological methods. The first type identified with genetic analysis has been added to a novel species (HAdV-G) and received the chronological number 52 [7].

Approximately one-third of the known types are associated with human diseases and different HAdVs have different tissue tropism that may include multiple tissues [8,9].

### 1.2. Epidemiology and Transmission

HAdV is a ubiquitous virus, which can be found all over the world. Infections can occur throughout the year, without a clear seasonality; the majority of infections are sporadic events, although epidemics may occur, especially in winter or early spring [8,10,11].

Over 80% of all diagnoses of HAdV infections are made in patients under 4 years of age because in this age group most children are not immune. Closed settings (such as schools, orphanages, military camps and hospitals) and immunocompromised patients are other populations with higher risk of becoming infected [8,12,13,14]. Measures for infection containment and detection of cases are thus crucial in institutionalized settings for preventing the spread of the virus [8,15] and in order to protect patients with immunodeficiencies, who are at greater risk of disseminated and severe disease [16].

The exact prevalence and incidence of adenoviral infections are unknown, since the infection is usually self-limiting and patients do not visit a general practitioner, or, if they do, the physician does not carry out microbiological investigations [10]. However, HAdV is estimated to be the causative agent underlying the 2–5% of all respiratory infections in the world [13].

The infectious process can occur in different ways, including droplets (inhalation or contact with the conjunctiva), fecal–oral transmission or through contaminated fomites in contaminated baths, pools, and ponds. HAdV can survive up to weeks on surfaces and is resistant to many disinfectants thanks to its non-enveloped structure. However, it can be inactivated by heat, formaldehyde and bleach. The incubation period is from 2 to 14 days [8,10]. Occasionally, infections during labor and delivery or with solid organ transplantation have been described [10].

### 1.3. Clinical Features

HAdV can cause different signs and symptoms, depending on the age of the patient, their immune status and the infecting HAdV species [10] (Table 1). Adenoviral infections account for 2–7% of respiratory illnesses and 4–20% of pneumonia [17,18]. In younger children, acute upper respiratory tract infections (URTIs) prevail, and often coexist with gastrointestinal (GI) symptoms [8]. In older children, URTIs are frequent, and pharyngoconjunctivitis, non-purulent swimming pool conjunctivitis, hemorrhagic cystitis, and mesenteric adenitis are often seen [10,19]. Myocarditis and neurologic involvement with febrile seizures, encephalitis, acute disseminated encephalomyelitis, and aseptic meningitis have been rarely reported [10].

The typical symptoms of airways involvement include fever, pharyngitis, sore throat and cough. In the case of lower respiratory tract infections (LRTIs), i.e., pneumonia and bronchiolitis, children usually have prodromes such as fever and myalgia with worsening cough and dyspnea, and they are unresponsive to antibiotic therapy [8,10,23,24]. Severe respiratory infections, requiring intensive care unit admission or invasive or non-invasive ventilation, are more apparent with HAdV type 7 and 8 and affect up to 15–20% of children with adenoviral LRTIs, mainly young infants and those with underlying conditions [12,24]. Up to 5–9% of severe adenoviral LRTIs have been reported to result in death [24,25]. In the case of pneumonia, the clinical presentation of HAdV is basically similar to that of bacterial infections (high-grade fever, poor general conditions, possible tachypnea or dyspnea, crackles on auscultation) and further investigation is required to ascertain the etiology, as discussed in the following paragraphs.

Severe or disseminated adenoviral disease can develop in groups at risk, particularly in severely immunocompromised children: patients with congenital immunodeficiencies (such as Severe Combined Immunodeficiency) and those undergoing allogeneic hematopoietic stem cell transplantation or solid organ transplantation [10,19,26]. In addition, lymphopenic patients showing <300 of lymphocytes per µL of peripheral blood, such as children receiving chemotherapy for acute lymphoblastic leukemia, may suffer from disseminated HAdV disease. Patients receiving alemtuzumab (anti-CD52 antibody), anti-thymocyte globulin, and the immunosuppressive treatment associated with graft-vs.-host disease are prone to invasive infection. The common point of these conditions is a profound lack of T cells, which are insufficient or whose function is severe impaired [7]. Patients with milder forms of immunodeficiency (later identified as selective IgA, IgG, Common Variable Immunodeficiency, etc.) are prone to Adenoviral infections but do suffer from a common and milder form of the disease [27].

### 1.4. Adenovirus Persistence and Reactivation

Due to the fact that it is capable of entering into different types of cells, HAdV has been found in lung cells, epithelial cells, in brain tissue, in intestinal lymphocytes and in T lymphocytes of tonsils and adenoids. HAdV can be internalized in cells without active replication, even in the absence of acute disease. The gastrointestinal tract represents the most important site of persistence in children. Infected mucosal lymphocytes in which HAdV persists in a subclinical state have been shown to be the source for viral reactivation during periods of severe immunosuppression, such as for stem cell transplantation [9,16]. The virus has shown the ability to replicate in epithelial gastrointestinal cells, suggesting that mucosal lymphocytes account for a reservoir of HAdV. As a consequence, in these patients, the rise of the number of copies in stools predicts the risk of HAdV viremia and disseminated infection [28].

Another common site of persistence is the adenoid tissue, from which asymptomatic shedding of the virus may occur. Although the majority of patients suffering from obstructive sleep apnea that harbor adenovirus in palatine tonsills and adenoids do not actively replicate the virus, it is suggested that these tissues may be implied in the spread of the virus among healthy individuals. The mechanism of reactivation seems to reflect previous observations: lymphocytes as permissive reservoirs, replication in epithelial cells, preceding potential viral dissemination [29].

The persistence of the infection in the airways can induce long-term sequela, such as bronchiectasis, and increased risk of protracted bacterial bronchitis, due to a chronic neutrophilic inflammation [8,30]; in addition, HAdV may be involved in mechanisms underlying the development of post-infectious bronchiolitis obliterans [31].

### 1.5. Immune Response

HAdV disease is controlled by both innate and adaptive immune responses. Briefly, the virus enters target cells via endocytosis, its infectivity is primed in endosomes by uncoating of the capsid and escape of endosomal membrane [32], then DNA enters the nuclear membrane through the Nuclear Pore Complex (NPC) [33,34]. At each step of host invasion, from receptor binding to DNA delivery to the nucleus, our immune cells (macrophages, dendritic cells, natural killer cells, and a range of B and T cells) can induce specific responses in order to impede the infection and to evoke defense reactions in other cells. One of the first mechanisms of mammalian cells defense is blocking the uncoating by defensins. α-defensins and β-defensins are produced both by immune cells (neutrophils) and epithelial cells in response to proinflammatory stimuli [35]. Human α-defensin 5 (αHD5) binds to the penton base fiber of HAdV (types A, B, C, E), stiffening the capsid and preventing the exposure of key viral products that are involved in endosome escape [36]. The failed escape from the endosome destines viral particles to degradation in lysosomes.

The complement system is a well-preserved mechanism in mammals. Components such as C1q, C3 and C4 opsonize the virus and promote endocytosis, irrespective of the receptors that ordinarily bind to the capsid for virus entry (such as Coxsackie-Adenovirus Receptor or integrins). In doing so, viral particles are directly delivered to the degradation process in lysosomes [37].

When the virus is coated with Immunoglobulin G, either as a single particle or immune-complexed, it enters the cells via endocytosis and enhances the recognition by the intracellular Fc receptor (TRIM21). The capsid fails to uncoat, and viral particles are then ubiquitinated and degraded by the proteasome [38].

Macrophage and other antigen presenting cells (APC) retain soluble receptors that recognize repetitive patterns of microorganisms, including HAdV. These receptors include toll-like receptors (TLR-9) that elicit secretion of gamma interferon (IFN-γ), tumor necrosis factor (TNF), interleukin-1 (IL-1), IL-2, and macrophage inflammatory protein [39]; these cytokines promote an antiviral activity and limit the amplification and spread of the virus [7,40]. The sensing of HAdV DNA particles that escaped ubiquitination or were mis-delivered through the nuclear pore complex and failed to enter the nucleus, triggers the cGAS/STING pathway and leads to the production of the aforementioned inflammatory cytokines [41]. For example, in the presence of low levels of HAdV antibodies, macrophages were shown to secrete proinflammatory molecules IL-1β and TNF-α without lysosomal damage, inflammasome activation or cell death [42]. Together with macrophages, natural killer cells are recruited and activated to destroy the cells infected by the virus [43].

Coming to the adaptive arm of the immune system, preclinical observations on the T cell response prove that these cells give a major contribution in counteracting the infection. CD4+ T cells mediate the production of antibodies by B cells and promote and maintain CD8+ T cells response thought CD40/CD40L, and IL-2 production in the proximity of dendritic cells [44]. CD4+ T cells inhibit viral replication in-vivo, and the effect was shown to be directly dependent on the number of T cells administered. Interaction between HLA-restricted CD4+ T cell clones and the target cell is needed for the antiviral effect, and it is enhanced in the presence of IFN-γ. Lysis of infected cells is then achieved with a perforin dependent mechanism [45]. The importance of the T cell response has been further highlighted in allogenic stem cell transplantation (SCT) recipients, in whom a profound T cells depletion increases the risk of disseminated HAdV infections with a fatal outcome. Conversely in immunocompetent individuals, HAdV-specific T cells are produced and able to recognize well-conserved peptides among HAdV serotypes [45], the CD4+ and CD8+ T cells against HAdV display cross-reactivity with different adenoviral species and types [46,47]. As a consequence, HAdV immunity in adults is carried out by cross-reactive helper and cytotoxic T cells generated by exposure during infancy and childhood and can be protective against most HAdV species [7]. On the other hand, B cells produce antibodies that are rather type-specific. As mentioned before, they are involved in the internalization and neutralization of HAdV preventing the uncoating needed for infection and can induce inflammasome activation in cells internalizing opsonized HAdV [42]. In SCT recipients, high titers of neutralizing antibody to a specific serotype of HAdV appeared to predispose to, instead of protect from, infection with that same serotype after transplantation [48], suggesting that a recent infection together with a longer memory of the adaptive response are fundamental. In other viral infections (such as influenza and viral hepatitis), the presence of the antibody itself is protective against the infection and even with HAdV in immunocompetent patients, antibodies were shown to give a certain grade of protection. Despite this latter observation, it is clear that the central role in prevention seems to be played by cell-mediated immunity acting together with the adaptive humoral response.

### 1.6. Diagnosis of Adenoviral Infection and Disease

In diagnosing HAdV infections, it should be emphasized that detection of HAdV is not always associated with acute disease. Indeed, proven disease is confirmed only in the presence of signs and symptoms and viral detection or histological confirmation in the appropriate location, but signs and symptoms compatible with HAdV infection without viral detection or histological confirmation are accounted as a probable disease.

Once diagnosed, HAdV infection can be further distinguished as local, systemic and disseminated [49]. Local infection is defined by either DNA or antigen detection or virus isolation in a specimen other than peripheral blood. The systemic infection definition relies on the same detection methods but HAdV is found also in peripheral blood. Disseminated disease with multiorgan-involvement can be diagnosed in the presence of at least two PCR-assays positive for HAdV-DNA in different specimens (biopsies, bronchoalveolar lavage, stools, aspirates, etc.) and in peripheral blood. These definitions for the diagnosis of HAdV-related disease have been developed for solid organ and allogeneic stem cell transplantation recipients, who may even undergo screening strategies for preclinical infections [7]. In immunocompetent patients, who tend to suffer from mild and localized infections, peripheral blood, stool, urine, bronchoalveolar lavage (BAL), nasopharyngeal aspirates, or swabs can be collected for testing for HAdV depending on the site of the suspected infection. Numerous techniques may be employed such as serology, point-of-care and laboratory-based antigen-detection methods, culture and hystopatology, nucleic acid-amplification methods (PCR), in situ hybridization, and immunohistochemistry.

The recognition of the typical histopathological changes of HAdV on target organ biopsies remains the gold standard [50,51] for the diagnosis of acute disease. However, these methods harbor some limitations. As HAdV affects mainly young and immunocompetent hosts, biopsies are not mandated and follow-ups are rarely warranted, clinicians should rather rely on more rapid methods and point-of-care testing.

HAdV cultures and isolation can be performed from any specimen [52] and do not give rise to misinterpretation due to the typical cytopathic effect produced by HAdV on permissive cells employed as growth medium. However, their sensitivity is influenced by sampling and transport conditions, different types may necessitate specific medium, and finally, up to 28 days are needed for results [16]. Due to its characteristics, culture is mainly employed for epidemiology or research purposes. In human lymphocyte cell cultures, almost all HAdV species induce clumping and cell rounding with intranuclear inclusions. The same cytopathic effect can be highlighted in vivo together with a broad spectrum of histopathological changes. For example, when acute pneumonia is observed, enlarged lung fields and hemorrhagic areas are often present, interstitial spaces show capillary leakage, and alveoli are filled with fluid and red blood cells. In biopsies, hyaline membrane formation, post infective bronchiolitis, and mononuclear infiltration is typical [8].

Another valuable tool is represented by a rise in two-sample serology [53]: a 4-fold rise in the IgG antibody titer is considered a positive response and indicates acute disease. However, two-sample serology needs at least two assessments separated by a reasonable amount of time; therefore, it warrants patients’ follow up and the need for a second sample to titer. The presence of adenoviral antigen in fresh stools found with gastroenteritis or diarrhea (in the absence of other causes of acute diarrhea) indicates acute infection, but the detection of HAdV in the stool of a patient with no symptoms, or symptoms other than gastroenteritis, may indicate viral shedding only [54]. Enzyme immunoassay tests are used mainly for stool samples. Direct fluorescent antibody tests and lateral flow immunochromatographic tests can be used in nasopharyngeal swabs or samples from the airways. Even though monoclonal and polyclonal antibodies are widely available for these latter uses, they do not seem to retain sufficient sensitivity to screen immunosuppressed patients [55].

Nowadays, molecular testing holds superior sensitivity and specificity [56]. Moreover, PCR assays have recently become widely available and a standard diagnostic tool [57,58]. Primers and probes for HAdV PCR assays are usually designed according to genes that encode for the hexon capsid protein (usually E1A region), which is well preserved throughout HAdV species [59]. Species that show genetic heterogeneity and whose E1A region differs from common types may still be detected thanks to the use of multiplex and nested PCR [60,61,62]; hence, PCR can be effectively applied to the whole blood as an effective screening method in SCT recipients for HAdV. In URTIs, commercially available kits are often claimed to detect, through multiplex PCR, a wide range of respiratory viruses, HAdV included. Rather, caution should be applied, as each assay has pathogen-specific sensitivity and is usually able to detect only a limited number of HAdV types, keeping a suboptimal sensitivity with commercially available kits [63]. Moreover, testing for adenovirus in the nasopharynx in the case of lower respiratory tract infections may not detect HAdV. In the last year, following the demand for the identification of the novel Coronavirus SARS-CoV-2, the routine implementation of PCR platforms is strongly advocated. Hence, point-of-care methods are readily available and do not need equipment or specialized training to be performed. They both give rapid results but can be successfully employed only in infection from common serotypes [56,64,65].

### 1.7. Treating HAdV Infection

Antiviral treatment is rarely indicated in previously healthy children with localized infection; hence, most of the recommendations regard allogeneic SCT recipients with systemic infections. The approaches may include prophylaxis, preemptive (pre-symptomatic treatment in the presence of HAdV) or therapeutic (symptomatic treatment in the presence of HAdV disease). Environmental hygiene measures are the first to be adopted in order to prevent outbreaks in transplantation units. Preemptive treatment is recommended by major guidelines in patients showing HAdV in stool samples (over a certain threshold, or rapidly rising) and at high risk of systemic dissemination. The rationale of the treatment is to slow down replication and diffusion of the virus allowing the reconstitution of a cellular immunity from the graft. Treatment options rely on tapering down immune suppression (when possible) or are based on antiviral agents or on isolation and transferring of HAdV-specific T cells from the donor [7] or a combination of the three. The drugs administered are intravenous cidofovir or oral brincidofovir, whose activity against adenovirus seems superior to ganciclovir and more feasible than ribavirin [66,67]. Cidofovir is a cytosine analog that inhibits DNA polymerase and displays activity against different HAdV types. Nephrotoxicity is the most feared side effect, including renal failure, Fanconi syndrome and interstitial nephritis. To avoid this side effect, it is usually given together with aggressive hydration and probenecid. Data on the efficacy of antiviral treatment are sparse and limited to case reports or case series, most of them assessed retrospectively, and controversy still exists on the befit of treatment. In one study, the viral load was assessed in blood after cidofovir in patients with and without T cells reconstitution. In the group without T cells reconstitution, 55% of patients showed stable plasma HAdV DNA levels after 2 weeks, 25% had an increase in viral load, and reduction without clearance was reported in the remaining 20%. Patients with T cells function showed an increase in viral load only in 9% of cases, with a rate of viral load reduction and clearance of 64% [68].

### 1.8. Potential Markers Based on Immunology

#### 1.8.1. Hematological Changes

Table 2 summarizes the main hematological changes during HAdV infections.

In the case of respiratory tract infections, HAdV causes a marked increase in CRP and procalcitonin together with WBC count and neutrophilic formula [71,72,73]. These markers are usually related to bacterial infection, promptly initiating antimicrobial prescriptions, but some distinctions based on the clinical picture should be made. In the case of an otherwise healthy infant with URTI and nasal discharge, presumptive diagnosis of bacterial infection can be made in the case of persistent symptoms (>10 days, with or without fever early in the disease), worsening disease course (late onset of fever, worsening of nasal discharge after initial improvement), or severe onset (fever >39 °C for 3 consecutive days and purulent discharge) [74]. With the suspicion of bacterial disease, antibiotics should be started empirically, covering the most probable bacterial agent aligned to the clinical picture, while cultures with antibiograms or other detection methods are awaited. This is performed in order to switch to targeted antimicrobial therapy, which may be withdrawn in the case of exclusive virus detection. With URTIs, watchful waiting prior to prescribing antibiotics may be used in the case of elevated CRP (found in point-of-care assays), since its elevation may be a surrogate marker of HAdV infection in a child that can be managed as an outpatient. CRP is often elevated in the case of prolonged adenoviral URTI.

WBC count is elevated during the acute phase of HAdV URTI, although this does not seem to differ between respiratory infection caused by other pathogens, including bacterial [75,76]. However, not all the HAdV species have been shown to cause elevation in WBC count [69]. What has been proven to characterize HAdV immune response is the neutrophilic prevalence [69,76,77,78] with neutrophil percentage over 40% in most studies, during the acute phase [79,80,81]. These findings may actually be confusing for at least two reasons. First, the neutrophil prevalence is found more frequently in bacterial infections rather than viral, leading often to empirical antibiotic therapy. Second, HAdV respiratory infections are found mainly in young patients, in which the lymphocyte prevalence in WBC count is characteristic, on the other hand, adolescents and adults usually show neutrophilic predominance in WBC count, and the neutrophilic shift caused by HAdV may not be noticed, as it represents normal predominance [82]. In conclusion, elevation of WBC count and neutrophilic predominance of the formula may be suitable markers of HAdV URTI in children.

Monocytes also vary during different phases and severity of HAdV infections. These are usually found elevated in the case of mild viral infections. Patients with pneumonia or URTIs with relatively severe presentation tended to have higher monocyte count than those with asymptomatic infection or healthy controls during the acute phases of infection [83], but initial monocytopenia has been shown as an independent predictor of respiratory failure in HAdV pneumonia in young adults [84,85]. Macrophages can be found early in the BAL of mice infected with Adenovirus pneumonia [86], where HAdV is readily internalized by tissue macrophages and generates a rapid inflammatory response. These observations imply that monocytopenia, although not specific, may develop as HAdV infection, which becomes more severe as the inflammatory process becomes uncontrolled or aberrant [85]. On the contrary, in the same study, monocytosis is found in more than half of the patients that did not need respiratory support. In the contest of relatively milder infections, such as URTIs, monocyte count elevation characterizes viral infections, such as infectious mononucleosis, and, among them, infections sustained by HAdV; on the other hand, in the ill appearing child with lower respiratory tract involvement, progressive monocytopenia should warrant careful evaluation and follow up.

Lymphocyte subpopulations are not studied routinely in patients with suspected infection from HAdV, but recently some promising markers have emerged. CD4+ T cells (T-helper) were found to be elevated in the case of silent HAdV 55 infections [83] and showed a tendency to higher values in the acute phase of URTIs compared to convalescence [75].

The complement system is a proteolytic cascade comprised of both soluble and membrane-bound proteins. It is an unspecific response to pathogens that can be activated by IgG and IgM antibodies (classical pathway) or that can activate spontaneously after contact with self and non-self-macromolecules. After the binding of an antibody to HAdV, circulating C1 attaches and cleaves complement proteins C2 and C4. The products of the cleavage combine to form the C3 convertase, which further cleaves C3 in C3a and C3b. C3b and C4b (a product of C4 cleavage) bind to the surface of the virion impeding infection progression; extracellular, opsonized HAdV virions can be recognized and internalized by receptors on cells (such as neutrophils and macrophage [87]). Intracellular HAdV bound to C4b are unable to unfold the capsid and escape endosomes, C3b-bound HAdV is degraded in the proteasome independent from TRIM-21 and activates the proinflammatory pathway [81,88,89].

**Figure 1:**
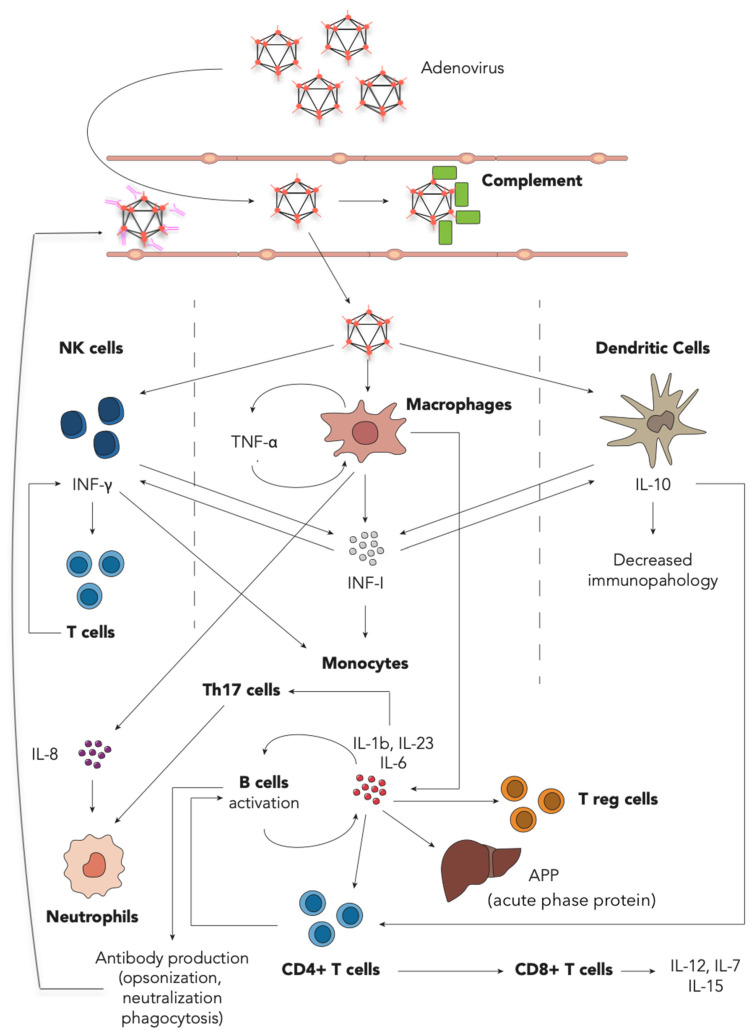
Adenovirus–host interaction during the first days of acute infection. On the top is the reported innate immune response starting with HAdV entry, blood stream invasion, complement activation and C3a release to promote opsonization and phagocytosis. Below, at target organ or tissue, interaction between HAdV and effector of the innate immune response is shown. The entering and sensing of HAdV by Natural Killer (NK) cells, macrophages and Dendritic Cells (DC) (through either penetration [34], phagocytosis of opsonized HAdV or CD46, Sialic Acid, integrins and MHC I interaction [90]) stimulates the production of Type I interferons (IFN-I). IFN-I is secreted in large amounts mainly by specialized DC, and, together with IL-12, IL-15, IL-18 (not shown) and drives the secretion of IFN-γ in NK cells. IFN-I enhances macrophage phagocytosis, activation and the expression of MHC I and MHC II [91]. The former enhances recognition of infected cells by cytotoxic T cells, and the latter, in antigen presenting cells, promotes the presentation and activation of the CD4+ T cell-response. Type I IFN and IFN-γ drive an overall antiviral response, promoting intracellular viral clearance and guiding the T cell response [91]. Moreover, NK cell-derived IFN-γ promotes further maturation and activation of DC and macrophages. IL-8 produced the course of the disease early and exerts its actions mainly on neutrophils, resulting in migration to the site of infection. TNF-α, an acute phase cytokine produced in large amounts by DCs, exerts proinflammatory actions on cells (cytokine production, expression of adhesion molecules, chemotaxis) and tissues (edema, cellular growth) partially counterbalanced by the IL-10 effect on immune-mediated tissue damage or immunopathology. At the bottom, both elements of the adaptive and innate immune response are shown. Proinflammatory cytokines (IL-6, IL-23 and IL-1β) are secreted by activated macrophages and DC and induce acute phase protein (APP) production in the liver and the adaptive cellular and humoral response [33]. Antigen presentation to CD4+ T cells (together with membrane co-stimulants and the presence IL-10) guides proliferation and differentiation of B cells, T helper 17 (Th17) cells and cytotoxic T cells (CD8+ T cells). Among other functions, B cells are committed to antibody production, CD8+ T cells induce apoptosis of infected cells and proinflammatory cytokine secretion (IL-12, IL-7, IL-15) [92], and Th17 could aggravate the inflammatory response by recruiting inflammatory cells and cytokine production. Conversely, Treg (induced during T cell response) plays a major role in counteracting immune-mediated tissue damage.

In an experimental model by Jie Tian et al., HAdV showed the ability to activate both pathways: in vitro, antibodies are needed to activate the classical pathway, but when injected in mice, antibodies played no significant role. The rise in plasma C3a, suggests the activation of complement is driven by an alternative pathway and other factors, including tissue damage [81]. A comprehensive description of the role of the cytokine and cell types involved in the HAdV immune response is provided in Figure 1.

#### 1.8.2. Cytokine Changes

Studies about the role of cytokines in the diagnosis of adenoviral infections in children and young adults are few and unfortunately burdened by a high level of heterogeneity in terms of different populations, clinical settings and diagnostic methods, and by wide time frames (over 20 years) during which they were conducted. A table of cytokines kit and analyzers used in the different studies is provided in the additional material (Appendix A, Table A1). The immune system varies greatly during childhood [93,94], and the eligibility of patients based on the typical span of the pediatric age range (0–14, 0–16 or 0–18 years) makes the comparison between groups difficult. Different HAdV species may elicit their own distinctive immune response and cytokine production, but not all studies make distinctions among types, and clinical criteria for infection severity are not standardized. The following paragraphs describe the main diagnostic and prognostic implications about cytokines during acute HAdV infection.

IFN-γ is detectable in the blood of more than 80% of patients during minor HAdV infections in children, such as URTIs or mild LRTIs [69], whereas low levels of IFN-γ characterize children with a worse prognosis [95]. These observations suggest that children possess a potent antiviral activity based on IFNs [83]. Even in young adults, IFN-γ is found elevated in the case of URTIs compared to minor infections [83], but interestingly, in young adults, higher values of IFN-γ are found in hypoxemic HAdV 55 infections, compared to non-hypoxemic cases, and are similarly found in cases of severe HAdV 55 pneumonia [84]. No differences in IFN-γ concentration were found in children (median age 4.1 years) from bacterial to viral agents [83] and between RSV and Influenza [72], but differences may be found in older patients (median age 21 years) between HAdV 55 and non-adenoviral pneumonia [84]. Although it has potential as a prognostic marker in some categories of patients, the role of IFN-γ as a surrogate marker for HAdV infection should be further studied.

Both IL-1β and TNF-α can be secreted by macrophages in response to viral infections in the infection site. In epithelial cell models, the production of IL-1β showed only a minor peak at the basolateral membrane, after 72 h of infection [96].

IL-1β is not found later in the course of the clinical disease [69,72,83,84], and its kinetic does not differ from bacterial to viral LRTIs, making it unsuitable as a marker of HAdV infection. As the role of tissue macrophages is to initiate prompt immune reactions in response to TLR and the pattern recognition receptors binding to HAdV activate the inflammasome early and drive the subsequent adaptive response, it might be supposed that these cytokines peak quite early in cases of HAdV infections. In fact, IL-1β levels may either spike before clinical presentation or represent only a local phenomenon throughout the infection, or may be marginally involved in the immune response against HAdV. Similarly, TNF-α does not seem to increase in serum during the early phases of both viral and bacterial infections and between different viral agents [72,84].

IL-6 is produced by a variety of cells such as vascular endothelial cells, mononuclear phagocytes, fibroblasts and activated T cells mainly in response to local secretion of IL-1 [97]. IL-6 is more elevated in cases of bacterial respiratory infections compared to viral or atypical agents [70,98]. Among viral infections, HAdV induced higher levels of serum IL-6 compared to Respiratory Syncytial Virus and Influenza virus, and this observation was greater among patients that required hospitalization [72]. In accordance, higher levels are observed in the case of severe HAdV infections compared to milder [73]; severe pneumonia showed levels up to 54.69 ± 38.22 pg/mL, common pneumonia 36.15 ± 23.30 pg/mL, and URTIs 30.78 ± 20.14 pg/mL. Moreover, during the acute phase, IL-6 levels were higher compared to convalescence [83] and healthy controls. Even in the case of HAdV URTIs in children, IL-6 was found elevated in more than 80% of cases during the acute phase [69] and decreased during convalescence [75]. IL-6 also showed its role as a potential HAdV marker in nasal swabs in the case of URTI when blood sample collection is not routinely indicated [99].

IL-8 was found to be produced by epithelial cells in response to HAdV [96]. Together with IL-6 and IL-10, IL-8 is found increased in the case of HAdV LRTIs in young adults compared to non-HAdV (24 vs. 16 pg/mL) [84], suggesting an overall more active cytokine-mediated inflammation; nevertheless, elevation of serum IL-8 in young adults does not predict disease severity as accurately as IL-6 and IL-10. On the contrary, in children, marked high levels of serum IL-8 were found (mean 1646 pg/mL in acute phase of HAdV URTIs) and higher values were associated with intensive care unit admission and need for respiratory support [75,83]. The IL-8 cytokine secretion in epithelial cells seems to peak later than IL-6 and 10 during infection and is maintained even in advanced stages [100]; moreover, the chemokine seems to be secreted in a distinct fashion depending on the side of the affected epithelium [96]. The basolateral edge of oriented respiratory epithelial cells infected with HAdV keeps the secretion of IL-8 far beyond the apical edge. Therefore, it can be then hypothesized that, even in URTIs, IL-8 in serum is found elevated for its preferential secretion coming directly from the airways and levels are found consistently high even in convalescence due to its prolonged production [75]. It should be noted that finding IL-8 elevation in nasal swabs of children may reflect the tip of the iceberg of the actual production of IL-8 and systemic levels, and this is supported by the fact that it represents a prognostic indicator for hospitalization [100]. IL-8 might have the potential to become a surrogate marker of HAdV infection in children: it is produced in the first 48 h from HAdV cell entry [96], and it is rather stable and can be assessed both in serum and in nasopharyngeal swabs giving different diagnostic and therapeutic implications [69,75].

In LRTIs, the levels of IL-10 peak in the first days of the infection and rapidly decline becoming undetectable during convalescence [70]. During HAdV URTIs, IL-10 is more elevated [75,76] in the early phases, suggesting a similar dynamic [75,83]. However, in LRTIs, IL-10 shows elevation in moderate and severe cases more than in mild and silent infections [83] and is positively associated with respiratory failure and hypoxemia [84]. Although reference values have been assessed only in small samples, and specifically for single HAdV types, there exists no cut-off definition in pediatric patients and studies remain concordant in describing IL-6 and IL-10 as indicators of both disease severity and progression [73,84].

In general, the aberrant release of multiple cytokines to eradicate the virus is seen also in cases of other viral infections such as with COVID-19. The histopathological examination of lungs of COVID-19 patients revealed a high concentration of proinflammatory cell subtypes (Th17, CD4+ T cells), and a serum increase in inflammatory cytokines (IL-6, IL-8, IL-1β) and chemokines is observed in severely ill patients compared to those with a milder disease [101]. Blood DC from older populations secrete higher levels of proinflammatory cytokines/chemokines, including IL-6, TNF-α [102], in response to interferon. The observation of severe HAdV pneumonia in children brings about concerns regarding a similar cytokine-derived mechanism. Moreover, the COVID-19-related multi-system inflammatory syndrome in children seems to elicit cytokine release in terms of IL-6, IL-10, IL-8 and TNF similar to patients hospitalized for URTIs caused by HAdV, suggesting a common immunological path to which the course of infection by these two agents may converge [75].

Table 2 summarizes these findings.

## 2. Conclusions

In this study, we reviewed the potential feasibility of markers that may unveil early respiratory infections caused by HAdV. A prompt recognition of the virus would limit the use of unnecessary antibiotics, which are often prescribed due to the frequent severity of adenoviral clinical presentations. However, at present there are no biomarkers allowing a precocious and definite diagnosis of HAdV infection. Implementation of rapid antigen detection kits would be advisable in the case of URTIs and might support the watchful waiting approach irrespectively of serum inflammatory markers. Monocyte elevation is a non-specific marker of viral infections in URTIs, but monocytopenia together with a prognostic marker for severe LRTIs should guide the suspicions of the clinician towards HAdV infections. Among inflammatory cytokines, IL-6, IL-8, IL-10 and IFN-γ showed precocious elevation in HAdV, but only IL-8 seems to retain enough stability along the disease course in children with URTIs.

In this review, the need for a comprehensive approach to the child with URTI is highlighted: it begins by outlining the seasonality, the epidemiology and the most susceptible population of HAdV infection. The need for placing an intravenous line for supportive therapy or antimicrobial administration is an opportunity for assessing serum markers that can hint at the diagnosis of HAdV infection. These include: WBC count with differential, IL-6, IL-8, IL-10, IFN-γ and HAdV specific antibody as a confirmatory test [53]. Patients that do not need hospitalization should not be given antibiotics in the case of URTI unless there is a prolonged course of disease or empiric bacterial detection. When blood samples are taken, the same markers could hint at the diagnosis of HAdV infection.

Given the success of the rapid antigenic test for SARS-CoV-2, we hope that the road pursued by the research may soon be followed for Adenovirus. For SARA-Cov-2, screening with rapid antigenic tests for SARS-CoV-2 have been rapidly developed, for cohorting of patients, in in-hospital settings, and outpatients for infection containment measures. Moreover, PCR techniques are specifically directed at targeting sequences highly conserved among variants of interest of SARS-CoV-2. Adenovirus is actually already globally spread and may reactivate, does not need strict cohorting of patients, is genetically heterogenous among different types (specific PCR primers have to be employed for different types), causes minor infections in immunocompetent patients and gives rise to unspecific symptoms (neither ageusia or anosmia). Therefore, rapid antigenic tests have been studied mainly for research purposes and multiplex PCR (for respiratory viruses) still harbors insufficient sensitivity for timely diagnosing Adenovirus infection to avoid a consistent number of inappropriate antimicrobials prescriptions. Conversely, more effort has been put into the screening of the population at risk for Adenovirus infections. Given the success, we hope that the road pursued by the research for SARS-CoV-2 may soon be followed for Adenovirus.

Further studies are needed to evaluate the approach to URTI caused by adenovirus mentioned earlier, with regards to cut-offs, sensitivity and specificity. Cross-sectional studies could compare these (and other) markers between HAdV and other viral or bacterial infections and possibly suggest a clinical score to be evaluated prospectively.

## Figures and Tables

**Table 1:** Common associations between general infection sites and HAdV types. Other HAdV species may also occur at the site of infection. Many HAdV species show great variability in their tissue tropism.

Localization	Clinical Manifestations	Most Affected Groups	Main Serotypes
Respiratory tract	Upper respiratory tract infection Lower respiratory tract infection	Pediatric patients Adult patients Outbreaks in hospitals, chronic care facilities, closed settings (e.g., military recruits)	Types 1–5, 7, 14, and 21 associated with small airways dysfunction and bronchiectasis in children, chronic obstructive pulmonary disease in adults
Gastrointestinal tract	Gastroenteritis, diarrhea Rare complications: hemorrhagic colitis, hepatitis, cholecystitis, pancreatitis	Young children Outbreaks in hospitals, chronic care facilities, closed settings (e.g., military recruits), patients with HIV [7]	Types 40 and 41
Eye	Epidemic keratoconjunctivitis Pharyngoconjunctival fever Non-specific conjunctivitis	Outbreaks in hospitals, chronic care facilities, closed settings (e.g., military recruits)	Types 8, 19, 37 (most common) Types 3, 4, 7, 11, 14 (less common) Types 53, 54, 56 associated with outbreaks [20]
Urinary tract	Dysuria, hematuria, hemorrhagic cystitis, renal allograft dysfunction	Hematopoietic stem cell transplant and solid organ transplant recipients	Types 11, 34, 35, 3, 7, 21 [8]
Disseminated disease	Disseminated	Hematopoietic stem cell transplant recipients, severe combined immunodeficiency (SCID) and patients undergoing antiblastic treatment.	Species A, B, C, D (most common) and F [7,21]
Rare manifestations	Encephalitis, meningitis Myocarditis, cardiomyopathy Mononucleosis-like syndrome Bronchopulmonary dysplasia Intestinal intussusception Sudden infant death	Mainly described in children [22]	Types 2, 3, 7 neurologic manifestations

**Table 2:** Principal serum markers and their clinical impact in diagnosis and management of HAdV infections in children and young adults. CRP = C reactive protein, HAdV = human adenovirus, URTIs = upper respiratory tract infections, humans. Between general infection sites and HAdV species. Other HAdV species may also occur at the site of infection, T CD4+ = T helper, T reg = T regulatory, IL = interleukin, TNF = tumor necrosis factor, IFN = interferon.

Marker	Advantages	Disadvantages	Limitations
CRP/procalcitonin	Early rise in serum in acute infections	May not differentiate HAdV and bacterial infections	Potential application in URTIs
White Blood Cells	Elevated in the acute HAdV infections	May not differentiate viral and bacterial infections	Observed only in some species [69]
Neutrophils	Elevated in HAdV rather than other viral infections	Typical also of bacterial infections	May not be suitable in older patients
Monocytes	Elevated in URTIs, prognostic significance	Not exclusive marker for HAdV	Prognostic value studied only in young adults
T CD4+	Elevated in URTIs and silent HAdV infections	-	Observed in some types, Not routinely assessed
T reg	-	-	Few and conflicting data, Not routinely assessed
IL-10	Early rise in serum in acute infections, prognostic significance	May not differentiate viral and bacterial infections [70]	Prognostic significance evaluated only in severe infections
IL-6	Elevated in HAdV rather than other viral infections, prognostic significance, may be assessed in nasal swabs	May not differentiate HAdV and bacterial infections	-
IL-8	Prognostic significance in children, may be assessed in nasal swabs, more stable along the disease course	Loss of prognostic value in young adults	-
IL-1β and TNF-α	-	Not exclusive marker for HAdV	Unclear course in HAdV infection
IFN-γ	Elevated in the acute HAdV infections	Not exclusive marker for HAdV in children	Prognostic value evaluated only in severe infections in young adults

## Data Availability

Not applicable.

## Appendix A

**Table A1:** Laboratory methods used for the assessment of inflammatory cytokines.

Authors	Kit Used	Analyzer and Software	Notes
Nakamura et al. [69]	MILLIPLEX MAP Human Cytokine/Chemokine Kit (Millipore, Billerica, MA, USA)	Luminex 100 TM analyzer (Luminex, Tokyo, Japan)	-
Fuchs et al. [70]	Human Cytokine/Chemokine Magnetic Bead Panel (Milliplex MAP kit Cat. # HCYTOMAG-60 K, Millipore Corp., Billerica, MA, USA)	Magpix Luminex (Luminex Corporation, Austin, TX, USA) and xponent software (version 4.2, Luminex Corp, Austin, TX, USA)	-
Moro et al. [99]	25-plex bead immunoassay kit (Biosource International, Camarillo, CA, USA)	Luminex 100 IS xMap multiplex system (Luminex Corporation, Austin, TX, USA) and Star Station V. 2.0 software (Applied Cytometry Systems, Sacramento, CA, USA)	Samples were stored at −80 °C until use, thawed at room temperature, and diluted 1:1 before analysis, according to the manufacturer’s instructions
Lim et al. [84]	Quantikine HS Human IL-6 Immunoassay, HS Human TNF-α, Human CXCL8/IL-8 Immunoassay, Quantikine Human IFN-γ and Human IL-10 Quant HS. ELISA kit, R&D, Minneapolis, MN, USA).	-	Assays were performed following the manufacturer’s instructions
Kawasaki et al. [72]	Enzyme-linked immunosorbent assay kits from Endogen (Endogen, Inc., Woburn, MA, USA)	-	-
Chen et al. [83]	Human cytokine kit (Milliplex, Catalog no. MPXHCYTO-60 K-16, Millipore, Billerica, MA, USA)	FLEXMAP 3D system (Luminex, Austin, TX, USA)	Assays were performed following the manufacturer’s instructions
Kleiner et al. [94]	Magnetic bead-based multiplex immunoassays (Bio-Plex) (BIO-RAD Laboratories, Milano, Italy)	Bio-Plex 200 reader and Bio-Plex Manager software	-
Biserni et al. [75]	MILLIPLEX MAP Human Cytokine/Chemokine Kit (Millipore, Billerica, MA, USA)	Luminex 100 TM analyzer (Luminex, Austin, TX, USA)	-
Mistchenko et al. [100]	ELISA kits for Human Cytokines from R&D Systems (Minneapolis, MN, USA)	-	-
Sun et al. [73]	-	-	Retrospective collection of IL-6 concentration
Nakamura et al. [69]	MILLIPLEX MAP Human Cytokine/Chemokine Kit (Millipore, Billerica, MA, USA)	Luminex 100 TM analyzer (Luminex, Tokyo, Japan)	
